# Drivers and Barriers to Improved Data Quality and Data-Use Practices: An Interpretative Qualitative Study in Addis Ababa, Ethiopia

**DOI:** 10.9745/GHSP-D-21-00689

**Published:** 2022-09-15

**Authors:** Hibret Tilahun, Biruk Abate, Hiwot Belay, Abebaw Gebeyehu, Mohammed Ahmed, Akiliu Simanesew, Wondimu Ayele, Afrah Mohammedsanni, Barbara Knittel, Yakob Wondarad

**Affiliations:** a John Snow Research and Training Institute, Inc., Addis Ababa, Ethiopia.; b Ethiopian Ministry of Health, Addis Ababa, Ethiopia.; c Addis Ababa Health Bureau, Regional Health Bureau, Addis Ababa, Ethiopia.; d Addis Ababa University, Addis Ababa, Ethiopia.

## Abstract

**Introduction:**

An objective of the Information Revolution Roadmap of Ethiopia’s Health Sector Transformation Plan was to improve health management information system (HMIS) data quality and data use at the point of health service delivery. We aimed to assess drivers of and barriers to improving HMIS data quality and use, focusing on key Information Revolution strategies including Connected Woreda, capacity building, performance monitoring teams, and motivational incentives.

**Methods:**

We conducted an interpretative qualitative study across all 11 health centers in 3 subcities of Addis Ababa, Ethiopia: Yeka, Akaki-Kaliti, and Ledeta. A total of 40 key informant interviews and 6 focus group discussions with a total of 43 discussants were conducted. We coded information gathered line-by-line and grouped responses under thematic codes as they emerged. Findings were triangulated and validated.

**Results:**

Our findings indicate that the main drivers of data quality and use at the point of service delivery were the use of the Connected Woreda strategy and its tools, capacity-building activities including mentorship, performance monitoring-team activities that led to active leadership engagement, and motivational incentives for data producers and users. Barriers to optimal data-use practices were the use of duplicative data collection tools at health facilities, under-developed health information system infrastructure, inadequate health information technician staffing and capacity limitations at the health facility level, insufficient leadership commitment, and unfavorable health worker attitudes toward data.

**Discussion:**

Improvements in quality and use of HMIS data at health facilities are expected to result in delivering better-quality health services to the community as data enable health workers to identify gaps in health care, fix them, and monitor improvements. Future investments should focus on strengthening the promising data-use practices, resolving bottlenecks caused by duplicative data collection tools, enhancing individual and institutional capacity, addressing suboptimal health worker attitudes toward data, and overcoming infrastructure and connectivity challenges.

## Abstract (precis)

The Ethiopia government’s implementation of strategies to improve data quality, as outlined in its Information Revolution Roadmap, has led to higher data quality and improved data use, but barriers to optimal data-use practices must be addressed to create a culture of information use.

## Teaser

**Key Findings:**

- Using the Connected Woreda strategy to assess health facilities’ status of data use, as well as capacity-building efforts and leadership engagement, was found to be useful for improved data quality and use.
- The use of performance management teams, which review data and monitor health facility performance, was the main driver in improving data quality and data-use practices at the health facilities.
- Infrastructure challenges and health workers’ negative attitudes and practices around data collection and use were barriers to data use.

**Key Implications:**

- Future investments to address health worker attitudes, human resource shortages, standardization of data registration tools, and infrastructure challenges, including district health information system version-2 system instability, are recommended.
- Continuing the commitment to the information revolution beyond the current health sector transformation plan period (2015–2020) is key to ensuring sustainability.

## INTRODUCTION

Data quality is a cornerstone of well-functioning health systems. Having sound and reliable information enables better resource allocation; more targeted care, policy development, and implementation; and more effective health education and training.^1^^–^^3^ However, data generated at peripheral levels of the health system are usually put in administrative reports and on shelves, rather than being used to improve health care.^4^ The extent to which decision makers use data in the decision-making process can depend on various behavioral and organizational factors.^5^ Some of the behavioral factors that influence data quality and use are a lack of a perceived benefit of the data to health,^6^ gaps in knowledge and skill to process and analyze data,^7^^–^^9^ and the absence of a “culture of information use.” Organizational determinants documented in the literature include lack of training, supervision, and mentorship^7^^,^^10^; limited availability of resources^9^^,^^11^; and inadequate leadership and coordination.^10^

In 2008, Ethiopia launched a national health management information system (HMIS) to standardize the system across the country—including harmonizing key indicators, data collection tools, reporting systems, data quality assurance mechanisms, and data-use systems and processes.^12^ Following the initiation of that effort, the country embarked on several initiatives to ensure the quality of data generated through the HMIS. These include building technical capacity, providing the necessary tools, conducting regular national data quality reviews and routine data quality assessments, using lot quality assurance sampling techniques, and digitizing the HMIS.^12^^,^^13^

To create a national commitment to enhancing the quality of data generated from the HMIS and to promote its use for decision making, Ethiopia’s government launched the “Information Revolution (IR)” roadmap in 2015. This initiative, as part of the first Health Sector Transformation Plan, aimed to cultivate a culture of information use in the health sector, digitize priority health interventions, and strengthen the governance of the HMIS.^12^ In line with this effort, in 2017, the government and the Bill & Melinda Gates Foundation launched the Ethiopia Data Use Partnership project as a joint initiative to support the implementation of Ethiopia’s IR Roadmap.^14^

The Information Revolution Roadmap aimed to cultivate a culture of information use in the health sector, digitize priority health interventions, and strengthen the governance of the HMIS.

Since then, the Ethiopian Ministry of Health (MOH) has applied different strategies to improve data quality and data-use practices. One of the key strategies is the Connected Woreda (CW), which aims to facilitate improvements in data quality and use. CW uses a tiered pathway along which facilities and districts progress to achieve greater standards in data quality and use.^15^^,^^16^ The CW assessment checklist helps to measure improvements in health information system (HIS) infrastructure, data quality, and data use by assessing health facilities and district health offices against a list of criteria (Table 1).^15^ Based on the assessment’s outcome, a team of health experts identifies gaps and devises solutions to address them.

**TABLE 1.** Connected Woreda Information Revolution Status Assessment Components, Addis, Ababa, Ethiopia, February 2022^16^

**Assessment Category**	**Assessment Criteria**	**Weight out of 100**
Health information system structure and resources	Fully equipped central medical record unit Health information system budget allocation Supportive supervision Capacity needs assessment Electronic information systems	30%
Data quality	Data quality assurance mechanisms in place (conducting lot quality assessment sampling) Report completeness (number of reports and content completeness) Report timeliness	30%
Data use	Performance monitoring team (presence and frequency of meeting) Review of key indicators (gap identification, root cause analysis, and developing and implementing an action plan) Feedback provided on performance Data visualization and dissemination Data-driven review meetings	40%

Based on IR Roadmap recommendations, the government, with the support of stakeholders and partners, rolled out a strategy to use performance monitoring teams (PMTs) in health facilities. These diverse teams—consisting of nurses, health officers, laboratory technicians, pharmacists, environmental health professionals, medical doctors, and midwives—meet at least once a month at the health facility to review HMIS data, assess data quality, and apply basic analysis techniques to prepare the data to inform decision making. Once the PMT identifies data issues and performance gaps, they are expected to conduct a root cause analysis and develop and implement a tailored action plan to remedy the underlying problems.^12^^,^^15^^,^^17^^,^^18^ In addition to this, the IR Roadmap recommended capacity-building efforts and incentive approaches to motivate data producers and users.^15^

We conducted an interpretative qualitative study in Addis Ababa, Ethiopia, to assess drivers of and barriers to data quality (completeness, accuracy, and timeliness) and use of routine data for decision making. In addition, we explored the strategies recommended by the IR that are believed to be effective in improving the quality and usability of HMIS data in Addis Ababa. Based on our findings, we propose recommendations to help data quality and data-use improvements.

## METHODS

### Study Setting and Design

Addis Ababa, the capital of Ethiopia, has a total projected population of 3,601,694 as of July 2021. The city is administratively divided into 12 subcities. Primary health services are provided mainly through health centers, each having an average of 60 technical staff covering an average population of 40,000–50,000.^8^

We conducted key informant interviews (KIIs) and focus group discussions (FGDs) among a diverse group of experts working in all 11 health centers of the 3 subcities namely Akaki Kality, Yeka, and Lideta. These 3 subcities were purposefully selected because they were implementing IR Roadmap-recommended strategies to improve data quality and use practices—with support from the Addis Ababa University School of Public Health (AAUSPH) in collaboration with the Addis Ababa Regional Health Bureau (AARHB).

According to the routinely collected data by AARHB and AAUSPH, the 11 health centers showed improvements across 3 major measurement criteria of the CW strategy: HIS structure and resources, data quality, and data use (Table 1).

We collected 2 rounds of data: first in June 2018, before the implementation of the IR strategies, and again in January 2020 to measure improvements due to the interventions. The assessment was done using the CW assessment checklist, which recommends assessing health facilities before and after the interventions to measure improvements (Figure). Another CW recommendation is to label the facilities as “emerging” for achieving <65% of the standards criteria, “candidate” for between 65% and 90%, and “model” for >90%.^16^

**FIGURE:**
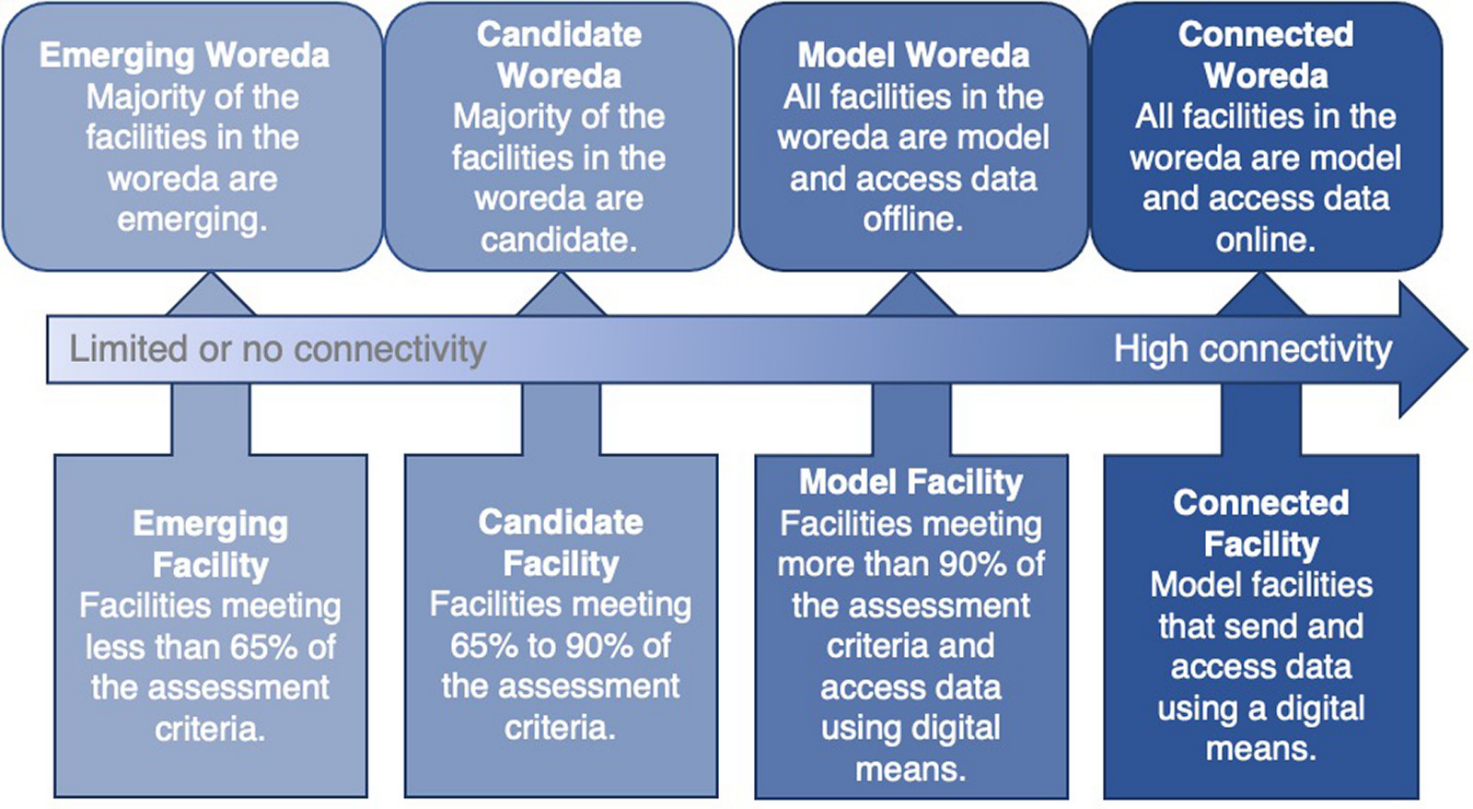
The Connected Woreda Pathway Used to Assess Health Facilities Data Use, Addis Ababa, Ethiopia

Accordingly, over the course of our assessment process, 5 health centers progressed to “model” status from “emerging” status; 4 progressed to “candidate” level from “emerging,” and 2 health centers remained at “candidate” level. Table 2 presents details of the assessment results.

**TABLE 2.** Connected Woreda Information Revolution Status Assessment Results, Ethiopia, February 2022

	**June 2018 Assessment (Pre-Intervention)**		**January 2020 Assessment (Post-Intervention)**	
	Standards Achieved – Total Score, %	Status Based on Connected Woreda Pathway	Standards Achieved – Total Score, %	Status Based on Connected Woreda Pathway
Akaki Health Center	22.7	Emerging	97.5	Model
Beletishachew Health Center	80.6	Candidate	82.0	Candidate
Hidasie Fire Health Center	58.6	Emerging	96.0	Model
Kality Health Center	33.7	Emerging	98.0	Model
Kilinto Health Center	24.9	Emerging	78.0	Candidate
Woreda 1 Health Center	60.2	Emerging	89.0	Candidate
Woreda 4 Health Center	54.1	Emerging	95.0	Model
Woreda 5 Health Center	59.9	Emerging	85.0	Candidate
Woreda 8 Health Center	55.9	Emerging	86.0	Candidate
Woreda 12 Health Center	48.5	Emerging	92.0	Model
Woreda 9 Health Center	68.3	Candidate	71.0	Candidate

### Study Participants

We included study participants from health facilities, subcity health offices, AARHB, and AAUSPH to represent the different levels of the health care system including academia. A total of 40 key informants including heads (n=6) or deputy heads (n=3); health information technicians (HITs) and monitoring and evaluation officers (n=9) and service providers (n=13) from health facilities; health information system (HIS) experts from the subcity health offices and AARHB (n=7); and a data-use expert from AAUSPH (n=1) were conducted. In addition, a total of 42 individuals participated in the 6 FGDs, including heads (n=1) or deputy heads (n=2); HITs and monitoring and evaluation officers (n=6) and service providers (n=21) from health facilities; and deputy heads (n=2), HIS experts (n=3) and health service program team leads/coordinators (n=7) from the subcity health offices. Experts were chosen based on their involvement in HMIS and data-use practices; newly hired experts (<6 months in the position) were excluded from the study.

### Data Collection Tool and Procedures

We developed a qualitative interview tool based on existing literature regarding barriers and facilitators to data quality and use. The interview guide was prepared in English, translated to Amharic, and pretested. Trained professional data collectors, with previous experience in similar data collection activities, collected the data. For each KII and FGD, 1 interviewer and 1 note taker were assigned. Interviews were conducted in the local language, Amharic. Each KII lasted about 30 minutes, while each FGD lasted about 2 hours and included 6–10 participants. The KIIs and FGDs were audio recorded and transcribed verbatim in Amharic, then translated to English for analysis.

### Data Analysis

We analyzed the data using a qualitative data analysis software package Atlas.ti 7. The interview transcripts were coded line-by-line and were grouped under thematic^19^ codes as they emerged. Two experts in qualitative study coded the transcripts resolving any disputes in consensus. We identified a total of 8 themes and 45 subthemes^19^ that directly related to the study objective. In presenting the data, we present relevant verbatim quotations to aid in the interpretation of the data.

We used the consolidated criteria for reporting qualitative research (COREQ)^20^ checklist as a reference in the design, analysis, and report write-up.^20^

### Ethics Approval

The Institutional Review Board of the Ethiopian Public Health Association reviewed and approved the study protocol and all ethical standards, including obtaining consent from the study participants. Confidentiality and privacy were maintained throughout the data collection process.

## RESULTS

Here, we describe data quality and data-use practices in the study areas and discuss the 4 key strategies of the IR Roadmap.

### Data Quality at Point of Health Service Delivery

Study participants consistently expressed improvements in data quality. They also stressed the necessity of data quality in creating trust in the data among users, thereby increasing its perceived value and use.

*We have so many changes compared to last year; for instance, when we see the registration and reporting system in delivery service, (in the past) data was not properly recorded and reported but currently, complete data is recorded.* —FGD participant

A best practice that study participants mentioned was the use of a PMT process that included identifying data quality and performance gaps, analyzing the root causes of these gaps, and planning and implementing tailored solutions to remedy performance and data quality gaps.

*We ensure the data quality through what is called a PMT team meeting on a monthly basis. The medical director writes feedback to each unit and recognizes those with good performances. There is also prioritization of gaps, discussion, and action plan will also be developed and integrated within the PMT logbook.* —KII participant

Study participants explained that experts from higher levels of the health system who conducted supportive supervision visits have helped to resolve challenges that compromised data quality. These challenges included a shortage of checklists and associated tools, interruption in connectivity for the District Health Information System-2 (DHIS2), and gaps in understanding data and indicators. Having regular structured supportive supervision and mentorship visits, guided by a checklist, was key to sustained data quality improvement, according to participants.

*Health facilities are showing improvement after the mentorship, especially with respect to data use and data quality and on the overall DHIS, there is a good improvement about all.* —KII participant

*Mentors identify gaps and provides feedback to staff on [the] spot and inform management.* —KII participant

Having regular structured supportive supervision and mentorship visits, guided by a checklist, was key to sustained data quality improvement.

Another technique that facilities applied to assure data quality was the use of the lot quality assurance sampling. Every month, after the collection of monthly data from all departments of the health facility, the HIT organizes the data and presents it to the PMT. The team then randomly identifies a few sample data and does a detailed review for accuracy and consistency, including cross-checking with the source data. This technique was integrated into the routine work of the health facilities to regularly identify and address data quality issues. If there were data quality and consistency gaps, the PMT provided feedback to those who work on the data and subsequently monitored improvements every month.

*We meet to evaluate the performance of data quality using [lot quality assurance sampling] to check both the report as well as the registration in departments. For [lot quality assurance sampling], we select indicators for this month to check data consistency and achievement versus targets and achievement is also compared with similar month in the last year. Through discussion, lower achievement indicators are identified for investigation and prioritized for improvement.* —FGD participant

### Use of HMIS Data at Point of Health Service Delivery

Overall, study participants explained that compared to the past, data use at the health facility level has improved. Within health facilities, different departments collect and use data to guide their work and track their performance.

*Generally, what we do is highly dependent on data; starting with forecasting of medication needs and estimating budget. For instance, if there are stillbirths, which are increasing in the health facility, we ask for justification. When the top 10 diseases are listed, it is based on data that we do analysis, vein analysis, and that essential drug lists are also defined by looking at which diseases are common.* —KII participant

Study participants emphasized the role of leadership commitment to improved data use. In health facilities where the leadership is committed to improving data quality and data-use practices, there was better performance in both, compared to health facilities where there was no meaningful leadership engagement.

*The medical director here needs to be praised for her commitment in making the performance monitoring team strong and active unlike other places; this is because of the medical director’s contribution.* —KII participant

Study participants consistently mentioned the lack of appropriate leadership as a bottleneck to improved data-use practices. In areas where there are poor data-use practices, health facility leaders are often not motivated to follow up on the HMIS.

*Let me just tell you one example, at one of the health centers, the leadership is very strong, they perform and responds to comments provided on the mentorship, the same mentor also supports another health center in the same catchment area, they received mentorship 4 times but there are still issues which were not solved yet. What we later identified as a cause for this was the lack of engagement in the leadership.* —KII participant

One of the challenges cited by study participants to properly collecting, organizing, and using data was poorly organized medical recording units with limited space and a lack of necessary computers, furniture, and shelves. This insufficient working infrastructure created concerns of potential vulnerabilities to data security and confidentiality. In addition, the instability of the DHIS2 system, due to connectivity issues and other related challenges (e.g., slow processing times to generate analysis), has been a challenge and created frustration among the health workers who fully depend on it for their day-to-day data-use work.

*There are challenges related with VPN and network breakdowns, and the DHIS2 also works through an online system and if this is not available, the professionals will not be able to do anything, you cannot talk about data use.* —FGD participant

*What the MRU (medical recording unit) were having as a challenge is lack of shelves and because of this patient medical records were being placed everywhere.* —KII participant

### Use of the CW Tools to Measure Baseline HMIS Data and Track Improvements

Study participants conveyed that the CW strategy tools, which were prepared to guide improvements in data quality and data-use practices, have helped them to assess the status of HMIS in the health facilities. These tools have also provided tailored support for facilities toward achieving model status by fulfilling the requirements.

The Connected Woreda tools have also provided tailored support for facilities toward achieving model status by fulfilling the requirements.

In contrast, because data collection tools (registers and reporting templates) other than the standard HMIS tools are being used, health workers have found it challenging to ensure consistency in the collected data. This issue created a burden among the health workers and further discouraged them to ensure data quality.

*There are many registration books to fill. For instance, in the OPT (Operation Triple A report) we usually find the mismatch between weekly and monthly report. When we tried to do it again, we find that there are many different registrations that are used as source document. For instance, there is the main registration itself, there is risk assessment registration at Outpatient Department (OPD) and there is PICT (Provider Initiated Counselling and Testing).* —FGD participant

### Use of PMT to Improve HMIS Data Quality and Use

According to participants, the PMT is the main driver behind the improvements in data quality and data-use practices at the point of health service delivery. Chaired by the head of the health facility, the PMT reviews the monthly routine health information to check for data quality and to monitor the overall performance of the health facility.

*Mainly, the performance monitoring team gives attention to data use which is the goal of a functional PMT. The members of the PMT are represented from all core processes and case teams and decision makers’ including the health center medical director. The PMT passes decisions based on data.* —KII participant

Study participants stressed the importance of the PMT’s role in promoting data quality given that the team reviews the health facility data during the last week of the month. Specifically, the team checks consistency, completeness, and other data parameters to validate its quality before it is used for further analysis and decision making. It is only after this stage that data from the health facilities were entered into the national HMIS using the DHIS2 software.

*PMT is a monitoring mechanism for all performed activities and services; especially activities performed with regards to the data management; we compare what is reported with the actual data recorded and compare consistency of the data within the reports, and for the quality components such as completeness and timeliness.* —KII participant

### Capacity Building on HMIS to Improve Data Quality and Use

Holistic capacity-building work in the form of classroom training and on-the-job mentorships through AAUSPH and AARHB have helped to build health facilities’ capacity and improve HMIS data quality and use.

Study participants confirmed that building the technical capacity of health workers on data analysis and interpretation—including how to collect, organize, analyze, and use data—was key for successful data-use practices at the point of health care delivery.

*Capacity building should be ongoing; it should be continuous as data is becoming more available and has to be strengthened. I want to stress that the activities related to data needs ongoing focus and capacity building to reach to the maximum level.* —KII participant

While study participants reported capacity limitations in advanced data analysis and interpretation, they can use basic Excel to organize, present, and use their data.

*We use excel for analysis and to organize a monthly, quarterly, and annual data, both service and disease reports. We prepare tables and charts to make it easily understandable by users.* —KII participant

According to participants, one of the challenges to data use is a shortage of human resources to manage health data at the point of health service delivery. Most health facilities are currently suffering from a HIT shortage as some have left their positions to upgrade their educational level.

*We have only 1 HMIS officer now and another 1 went for higher education, and we cannot recruit a replacement. We have no option except waiting until she completes her education, but this has influence on data use work, which means the data-use activities will be handled by 1 expert.* —KII participant

*We have challenges on quality of data in each department due to work overload. There is too much work in this health center. Shortage of human resource and infrastructure limitations are our challenges.* —FGD participant

### Use of Incentives to Improve HMIS Data Quality and Use

Participants reported that health workers were more focused on providing care for patients than on properly recording and reporting it. Findings suggested that a key challenge was posed by health workers’ unfavorable attitudes/perceptions and low level of commitment toward properly collecting, reporting, organizing, and using the data they collect to inform service delivery and improve access to quality services.

*Some professionals do nothing more than providing the medical service, and that is it; … they need training to change their skill and attitude.* —KII participant

*Health worker’s attitude towards data is not good enough; for example, when we check the registration book it has serial numbers that is not recorded in most of the time but has its own value; even summary sheets are not recorded …health worker don’t value it due to lack of awareness.* —FGD participant

Health workers’ unfavorable attitudes/perceptions and low level of commitment toward properly collecting, reporting, organizing, and using the data to inform service delivery and improve access to services posed a key challenge.

Study participants suggested that incentives were needed to motivate changes in health worker attitudes and practices around data collection and use. Some health facilities have used non-monetary incentives, like providing certificates, publicly recognizing, and offering training opportunities to high-performing health workers. Study participants agreed that using incentives as a strategy has a key role in motivating health workers to value HMIS data, but the incentives need to be contextually designed and implemented.

*When I found my name posted as ranking 1st (as best performer in data), I was motivated.* —KII participant

## DISCUSSION

We assessed data quality and data-use practices at health centers in Addis Ababa as well as the perceived effectiveness of CW, PMT, capacity-building, and incentive strategies implemented to address barriers to improving HMIS data quality and use for decision making. We used a qualitative methodology to gain a deeper understanding of the strategies including the drivers of and barriers to their effective implementation to impact data quality and use.

The role of the PMT in promoting data quality and use in the health centers was encouraging, and we recommend further study to gather enough evidence of the mechanisms PMTs applied. The importance of review forums like the PMT in promoting data quality and use has been highlighted in other studies as well.^5^^,^^21^ Further efforts that help to optimize the functionality of the PMT, including using digital tools, should also be considered.

Leadership engagement has also been found to be a key driver to improving data quality and data-use practices at the point of health service delivery.^10^ Participants mentioned leadership engagement as a key factor for improvements in health centers with promising data-use practices, while they saw the lack of leadership as the main barrier in health centers where improvements were lacking. To benefit from this, the MOH, regional health bureaus, and partners should embark on a consistent sensitization of health facility leaders to generate the needed attention from leadership. In addition, designing capacity-building training specifically for leadership is also recommended.

A significant barrier to improving data quality and use was the lack of uninterrupted access to the routine health information data, caused by HMIS infrastructure challenges, such as inconsistent and limited internet connectivity. Reliable internet connectivity and functional computers are expected to improve timely access to quality data, which ultimately will improve the quality of care. Chaudhry et al. documented this issue by showing health information technology was associated with improved quality of care by increasing adherence to guidelines, enhancing disease surveillance, and decreasing medication errors in addition to gaining efficacy.^22^ Another study conducted in Ethiopia stated that lack of infrastructure—such as electricity, power generators, and computers—influenced the generation and use of health data at the facility level.^11^ Lack of HMIS resources were also reported to influence HMIS data quality in low- and middle-income countries.^23^

Study participants recognized the value of the capacity-building work done on data quality and data use. This finding is supported by literature emphasizing the need for building technical capacity at lower levels as an essential prerequisite to facilitating evidence-based decision making.^13^ Thus, we encourage the government and stakeholders to continue such capacity-building efforts. In addition, a recent scoping review revealed the importance of interventions that combine technology enhancement and capacity-building activities, along with a data quality assessment and feedback system in improving data quality.^24^ Another study also documented that training and capacity-building activities such as mentorship and supervision were associated with improved data quality.^25^ We recommend strengthening need-based trainings, supportive supervision, and onsite mentorship universally in all parts of the country.

Revisiting the HITs staffing structure and their availability at health facilities is also essential. The MOH can work with local universities to address this gap so that capacity building leads to sustainable improvement. A recent publication on HIS professionals in Ethiopia indicated a significant shortage in health systems professionals, which is a major bottleneck to ensuring data quality and data use.^26^

Motivational interventions and appropriate incentives that address health workers’ behavior and attitudes toward HMIS can enable collection, reporting, and use of quality data from the source.^11^^,^^27^ A study done in rural South Africa associated data quality problems with reduced level of health worker motivation,^28^ which is indicative of the need to address motivation-related challenges.

Motivational interventions and appropriate incentives that address health workers’ behavior and attitudes toward HMIS can enable collection, reporting, and use of quality data from the source.

Another critical challenge to improving data quality and use practices is the availability and use of registers that are not approved as standardized HMIS tools. Accordingly, we recommend that the government take bold action to weed out unstandardized HMIS registration tools and put a system in place to prevent the introduction of similar tools in the future.

Overall, study participants consistently confirmed improvements in HMIS data quality and use practices due to the implementation of the IR strategies, which is a promising sign of progress toward creating a culture of information as recommended by the national IR Roadmap. Once this cultural shift has occurred, improved date-use behaviors and practices in the health facilities will become more sustainable. Supporting the findings of the current study, previous national data quality reviews have also indicated improvements in HMIS data quality and use.^13^^,^^29^

## CONCLUSION AND RECOMMENDATION

In summary, participants cited Ethiopia’s IR strategies including capacity building in HIS, leadership engagement, establishment of PMT at health facilities, use of motivational incentives, regular mentorship visits, and application of the tools and strategies of the CW strategy as facilitating factors for improved data quality and use. In contrast, use of duplicate data collection tools, infrastructure-related challenges, lack of leadership commitment, limited technical capacity of HITs and health workers, inadequate staffing structure for data management at the health facility level, and unfavorable health worker attitudes toward data were cited as major barriers of optimal data quality and use. To strengthen data quality and use in Ethiopia, we recommend future investments to address health worker attitudes and infrastructure challenges, including DHIS2 system stability.

## Notes

**Peer Reviewed**
